# Deep Inspiration Breath Hold: Techniques and Advantages for Cardiac Sparing During Breast Cancer Irradiation

**DOI:** 10.3389/fonc.2018.00087

**Published:** 2018-04-04

**Authors:** Carmen Bergom, Adam Currey, Nina Desai, An Tai, Jonathan B. Strauss

**Affiliations:** ^1^Department of Radiation Oncology, Medical College of Wisconsin, Milwaukee, WI, United States; ^2^Department of Radiation Oncology, Feinberg School of Medicine, Northwestern University, Chicago, IL, United States

**Keywords:** deep inspiration breath hold, breast cancer, radiation, active breathing control, real-time position management, heart, cardiac irradiation

## Abstract

Historically, heart dose from left-sided breast radiotherapy has been associated with a risk of cardiac injury. Data suggests that there is not a threshold for the deleterious effects from radiation on the heart. Over the past several years, advances in radiation delivery techniques have reduced cardiac morbidity due to treatment. Deep inspiration breath hold (DIBH) is a technique that takes advantage of a more favorable position of the heart during inspiration to minimize heart doses over a course of radiation therapy. In the accompanying review article, we outline several methods used to deliver treatment with DIBH, quantify the benefits of DIBH treatment, discuss considerations for patient selection, and identify challenges associated with DIBH techniques.

## Introduction and Background

Although it is well known that radiation therapy for breast cancer has both local control and survival benefits (1, 2), many have questioned whether the associated toxicities and resulting mortality may actually negate some of the survival benefit (3, 4). It has been hypothesized that this mortality is specifically related to cardiac mortality, and studies have demonstrated that patients who receive radiation therapy for left-sided breast cancers have increased cardiac mortality (5, 6). In fact, mean radiation dose to the heart correlates with rates of both cardiac deaths (7, 8) and coronary events (8, 9). While the risk of cardiac toxicity is also influenced by other factors, such as the patient’s baseline cardiac risk, as well as cardiotoxic chemotherapy (10–12), these effects may not be synergistic (9). The risk of heart disease and coronary events is estimated to increase 4–7% for each 1 Gy in mean heart dose, and there does not appear to be a minimum dose threshold below which there is no risk of cardiac events (9, 13). Additionally, while irradiation of internal mammary chain (IMC) lymph nodes is still controversial (14, 15), more recent studies highlight the potential benefits of regional nodal irradiation (16, 17), which may increase the incidence of nodal irradiation in the future. As IMC irradiation is known to increase the heart dose when compared to whole breast or chest wall (CW) alone treatment (18), reducing cardiac dose and associated toxicity from radiation therapy may become even more important.

The studies which remain the basis for cardiac toxicity from radiation are from the pre-three-dimensional (3D) conformal radiation therapy era (4, 5, 19, 20), and advances in radiation therapy since then have decreased the mean heart dose (4, 6, 19, 21). Some of the techniques which have been shown to decrease heart dose include both prone breast radiation therapy, where the patient is simulated and treated, lying on the abdomen to pull the breast away from the heart (22), as well as proton therapy (23, 24). Of note, while modern techniques such as intensity-modulated radiation therapy (IMRT) decrease the volume of heart that receives high doses, a larger volume may receive lower doses, due to low-dose spray associated with the technique (25).

**Figure 1:**
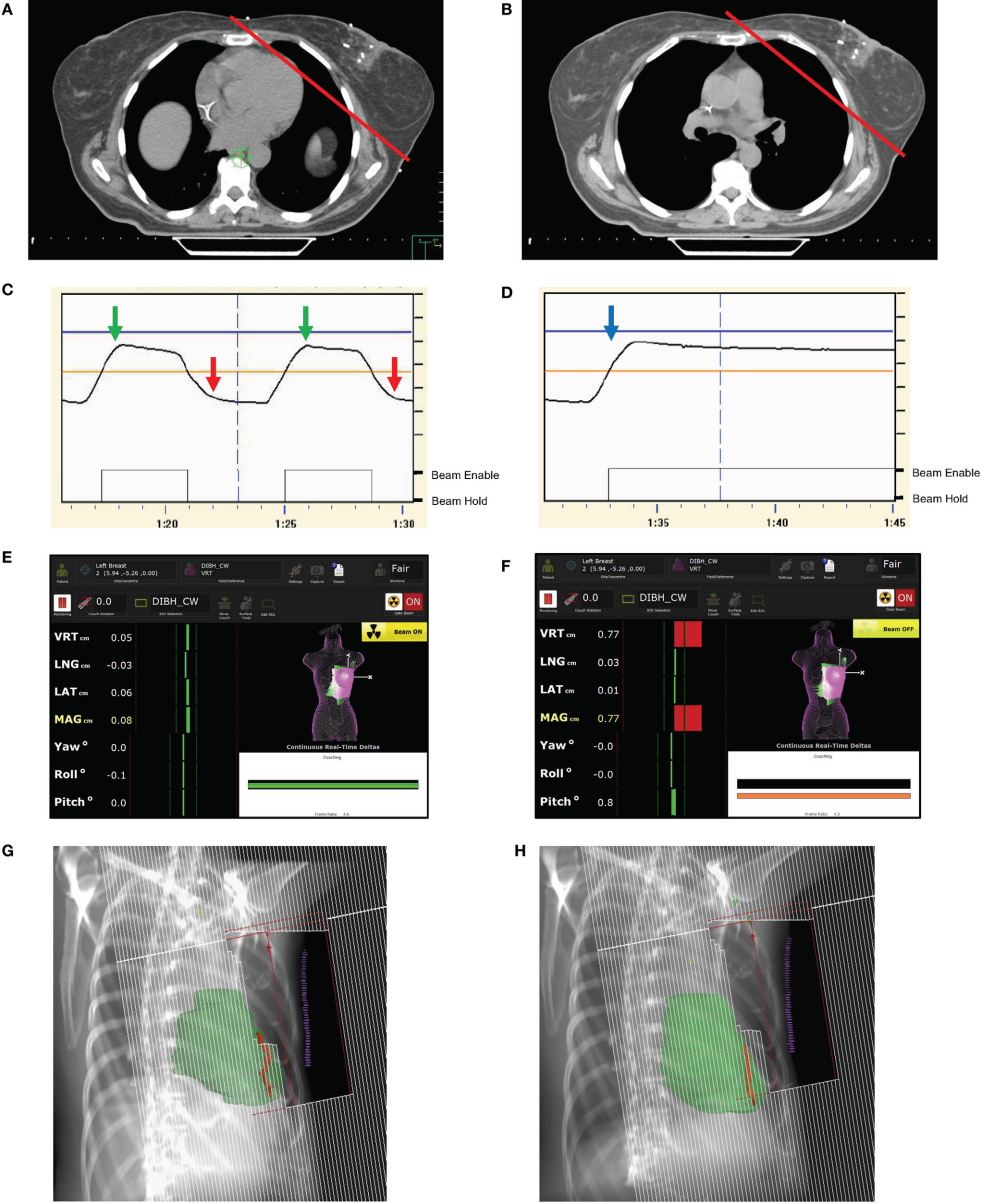

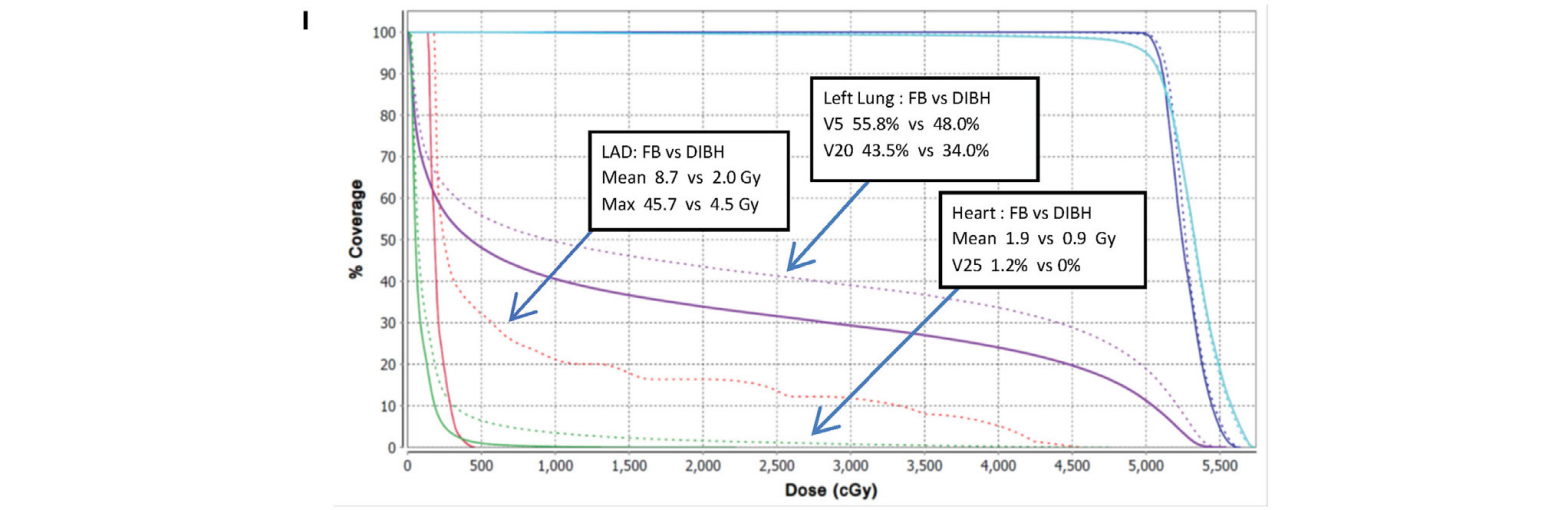
Deep inspiration breath hold (DIBH) techniques and examples of anatomic and dosimetric advantages. **(A,B)** Axial CT slices from the same level of the breast in free breathing **(A)** and DIBH **(B)** CT scans. The red line indicates the tangential radiation field used for whole breast radiation treatment. Note that the heart is easily excluded in the DIBH scan. **(C,D)** DIBH respiratory tracings and real-time patient monitoring (RPM) using optical tracking at inspiration (green arrow) and expiration (red arrow) during free breathing. **(D)** RPM tracings confirm appropriate chest excursion during DIBH, with a stable breath hold tracing. After inspiration (blue arrow) with DIBH, the beam would be on during the sustained breath hold. **(E)** AlignRT screenshot illustrates a reference surface on the chest wall (CW) used for alignment with a region of interest (left breast), which is matched during surface tracking, and with DIBH, when the left breast is within the preset thresholds, as indicated by the dynamic green bars on the side of the panel, the treatment beam is enabled. **(F)** With expiration or any movements of the left breast outside of tolerance, the green bars turn red and the radiation beam is held. This process continues until the whole radiation dose is delivered daily. **(G,H)** Digitally reconstructed radiographs from a patient in during free breathing **(G)** and voluntary DIBH (vDIBH) **(H)** demonstrating the favorable shift in heart (green) and left anterior descending artery (LAD) (red) positions in relation to CW tangent with vDIBH. **(I)** The dose volume histogram comparison of free breathing and vDIBH three-dimensional conformal radiation therapy plans for patient in **(G,H)** treating the left CW to 50 plus a 10 Gy boost, and regional lymph nodes including the supraclavicular and internal mammary chain nodes to 50 Gy. The organs are at risk in the vDIBH plan (solid lines), including the heart (green lines), left (Lt) lung (purple lines), and LAD (red lines), receive decreased doses when compared to the free breathing plan (dotted lines). The mean, max, and/or volumetric doses for these organs are provided in the boxes.

Another technique that can be used to decrease heart dose is deep inspiration breath hold (DIBH). The technique is based upon the observation that during inspiration, the flattening of the diaphragm and expansion of the lungs pulls the heart away from the CW. During both simulation and treatment, the patient takes a deep breath and then holds it for a period of time during which radiation is administered. This allows for a decrease in radiation dose to the heart (Figures 1A,B) (26). While DIBH can be used alternatively to prone breast irradiation (27), the two techniques can and have been used in conjunction (28, 29).

## DIBH Methods

Currently, there are two very commonly used techniques for DIBH, voluntary DIBH (vDIBH), and moderate DIBH. Moderate DIBH is a technique whereby devices known as active breathing control (ABC) devices are used (30) (e.g., ABC from Elekta, Stockholm, Sweden). These devices typically utilize a spirometer which allows for monitoring of air flow throughout the respiratory cycle and stopping air flow at a set threshold volume, causing the patient to hold their breath to maintain this volume (30–32). This has not only been shown to decrease the heart and left anterior descending artery (LAD) dose (Table 1), but has also proven to be very reproducible, which is always a concern when using motion management for radiotherapy. Specifically, one study demonstrated that ABC devices could reduce the set-up error to less than 2 mm, and in some cases, even 1 mm. Hence, a potential benefit of the ABC device is its ability to decrease the variability associated with the procedure, both within a fraction and between separate fractions.

**Table 1:** Reduction of mean LAD and heart doses in studies examining free breathing versus DIBH techniques for breast cancer radiation treatment.

Study	DIBH method	# Patients	Area(s) treated	Mean LAD dose (Gy)			Mean heart dose (Gy)		
				FB	DIBH	Reduction	FB	DIBH	Reduction
Stranzl and Zurl (33)	voluntary DIBH (vDIBH) (RPM)	22	Breast/CW ± boost	–	–	–	2.3	1.3	44%
Stranzl et al. (34)	vDIBH (RPM)	11	Breast/CW + IMC LN	–	–	–	4.0	2.5	38%
Borst et al. (35)	vDIBH (other)	19	Breast/CW ± boost	11.4	5.5	52%	5.1	1.7	67%
Johansen et al. (36)	vDIBH (RPM)	16	Breast	–	–	–	6.5	2.5	62%
McIntosh et al. (37)	vDIBH (RPM)	10	Breast	Not reported	Not reported	43%	Not reported	Not reported	48%
Vikstrom et al. (38)	vDIBH (RPM)	17	Breast	18.1	6.4	65%	3.7	1.7	54%
Hayden et al. (39)	vDIBH (RPM)	30	Breast + boost	33.7	21.9	35%^e^	6.9	4.0	42%
Hjelstuen et al. (40)	vDIBH (RPM)	17	Breast + SCV + Ax + IMC LN	25.0	10.9	56%	6.2	3.1	50%
Wang et al. (41)	ABC	20	Breast	20.0	5.9	71%	3.2	1.3	59%
Bruzzaniti et al. (42)	vDIBH (RPM)	8	Breast	9.0	2.7	70%	1.7	1.2	29%
Lee et al. (43)	vDIBH (RPM)	25	Breast	26.3	16.0	39%	4.5	2.5	44%
Mast et al. (44)	ABC	20	Breast	18.614.9	9.66.7	48%^a^55%^b^	3.32.7	1.81.5	45%^a^44%^b^
Nissen and Appelt (45)	ABC	227^c^	Breast/CW ± SCV + Ax LN	–	–	–	5.2	2.7	48%
Reardon et al. (46)	vDIBH (RPM)	10	Breast	2.5	1.8	29%^f^	1.6	0.9	45%^f^
Swanson et al. (47)	ABC	87	Breast/CW ± SCV + Ax LN	–	–	–	4.2	2.5	40%
Bolukbasi et al. (48)	vDIBH (RPM)	1010	Breast	1.75.0	0.84.0	53%^g^20%^h^	1.74.9	0.73.7	59%^g^25%^h^
Comsa et al. (49)	ABC	2030	Breast ± boostBreast/CW + SCV + Ax LN	––	––	––	3.14.5	1.22.1	61%53%
Osman et al. (50)	vDIBH (RPM)	13	Breast + SCV + Ax + IMC LN	––	––	––	9.05.8	5.04.1	44%^a^29%^b^
Verhoeven et al. (27)	vDIBH (RPM)	17	Breast	30.9	22.4	28%	3.5	1.6	54%
Eldredge-Hindy et al. (51)	ABC	86	Breast ± boost ± SCV + Ax ± IMC LN	–	–	–	2.7	0.9	67%^d^
Joo et al. (52)	vDIBH (RPM)	32	Breast/CW ± SCV + Ax	40.8	23.7	42%	7.2	2.8	61%
Mulliez et al. (28)	vDIBH (RPM)	12	Breast	17.6	10.9	38%	4.0	2.2	45%
Rochet et al. (53)	vDIBH (other)	35	Breast/CW ± SCV + Ax + IMC LN	14.9	4.0	73%	2.5	0.9	64%
Tanguturi et al. (54)	vDIBH (AlignRT)	146	Breast/CW ± SCV + Ax ± IMC LN	–	–	–	2.6	1.4	46%
Wiant et al. (55)	vDIBH (other)	25	Breast	–	–	–	3.0	1.4	53%
Yeung et al. (56)	vDIBH (other)	20	Breast/CW ± SCV + Ax + IMC LN	13.6	4.1	70%	2.6	1.3	50%
Walston et al. (57)	vDIBH (AlignRT)	78	Breast ± boostCW ± boost ± SCV + Ax + IMC LN	––	––	––	1.35.1	0.93.6	31%29%
Lawler and Leech (58)	RPM	28	Breast/CW ± SCV	10.9	5.2	52%	1.8	1.2	33%
Kunheri et al. (59)	ABC	45	Breast	13.2	6.1	54%	3.1	1.6	52%
Mohamad et al. (60)	ABC	22	Breast/CW + SCV + Ax + IMC LN	21.3	9.4	56%	5.8	2.2	62%

*ABC, active breathing coordinator; Ax, axillary; CW, chest wall; DIBH, deep inspiration breath hold; Gy (gray); FB, free breathing; IMC, internal mammary chain; LAD, left anterior descending coronary artery; LN, lymph nodes; RPM, real-time positioning management system; SCV, supraclavicular; IMRT, Intensity modulated radiation therapy*.

*^a^3D conformal radiation therapy*.

*^b^IMRT/VMAT*.

*^c^227 left-sided (144 received DIBH; 83 received FB treatment)*.

*^d^Median values for mean doses*.

*^e^LAD planning organ at risk volume (PRV)*.

*^f^FB-IMRT versus 3D-DIBH*.

*^g^FB versus DIBH forward-planned IMRT*.

*^h^FB versus DIBH inverse-planned IMRT*.

Alternatively, patients can undergo vDIBH, where respiratory motion is monitored, and the patient is instructed to hold his or her breath at certain points in the breathing cycle. One example of this technique is the Varian RPM (real-time position management) system (Varian Medical Systems, Palo Alto, CA, USA), where a device is placed on the patient’s chest and vertical displacement throughout the respiratory cycle provides surrogate data to create a tracing of the patient’s breathing (Figures 1C,D). With this technique, the patient is coached and must voluntarily hold their breath. The treatment beam can be gated so that treatment is stopped when the breathing signal falls outside a preset threshold. This type of gating in DIBH, in which the beam is turned off only in the event that the breath hold is out of target range, should be differentiated from standard respiratory gating, in which the patient is breathing free and the beam is repeatedly turned off during a predetermined portion of the respiratory cycle. Unlike DIBH, respiratory gating at free breathing is not usually an effective method for cardiac sparing because at no point in the standard respiratory cycle does the heart move dramatically away from the breast or chest wall. It appears that vDIBH is quite comparable to ABC DIBH on multiple levels. The UK HeartSpare study (61) compared the two techniques using a crossover study, where patients initially received treatment with one DIBH technique for one half, followed by the other technique for the other half of their therapy. The study noted similar overall treatment times, but found that vDIBH was associated with decreased time for both simulation and daily setup. Additionally, both patients and therapists endorsed greater satisfaction with the vDIBH technique (61). In fact, a study by Eldredge-Hindy et al. noted that 18% of the 112 patients enrolled in their study did not tolerate the ABC technique (62). All of these studies, along with the fact that vDIBH can be executed relatively cheaply (61, 63), question whether ABC is necessary for DIBH.

Optical tracking systems are more advanced tools (e.g., AlignRT, Vision RT Ltd., London, UK; Sentinel, C-RAD, Uppsala, Sweden) for DIBH (64), because RPM was shown to have inferior correlation with the target position, determined *via* MV cine imaging (65). In optical tracking systems, stereovision is used to reconstruct the 3D surface of the patient, visualizing the alignment of the reference surface and the reconstructed surface at the region of interest to provide real-time position monitoring (Figures 1E,F) (65–67). Betgen et al. (68) performed vDIBH using an optical surface tracking system. After set-up corrections, variations in chest excursion for each breathhold within a treatment fraction and within a treatment field were found to be very small, demonstrating reliable geometry of the CW (68) during a DIBH treatment. Another study also demonstrated high patient setup accuracy with the use of optical surface imaging (69). Similarly, when magnetic sensors were affixed to the thorax to measure chest excursion during DIBH, the SD of the amplitude of chest motion for each patient was <3 mm, indicating that the magnitude of inspiration could be reliably reproduced (70). Additionally, other manual checkpoints during the treatment, such as positioning of tattoo markings (61, 71) and image guidance (35) both ensure proper setup. More typically, a combination of the above-mentioned techniques is utilized (63).

For DIBH treatment, at least two CT scans corresponding to free breathing and DIBH have to be acquired during simulation. Patients are coached to breathe through their nose instead of mouth. The patient contours on the two CT scans will be used to match the corresponding daily patient surfaces from the optical tracking system during treatment setup for assuring that patients reach the same level of breathhold as that during simulation. When treating the left-sided breast patients with nodal involvement using DIBH delivery, both nodal and breast fields should be delivered at breathhold due to the matchline between nodal and breast fields. Feathering of the matchline may not be necessary during treatment because a 3 mm threshold of DIBH position uncertainty is usually applied in the surface tracking systems. The number of field-in-fields should be minimized (preferably <4 fields) for more rapid treatment delivery. This is one reason why IMRT planning is not often combined with DIBH delivery. Some types of bolus may not be tracked well by the optical tracking system. Wet towels may be used to replace the more rigid bolus during DIBH delivery (72). Alternatively, a conformal brass bolus can be used and cloth tape can be placed over the bolus to increase contrast for the optical tracking system.

## Advantages of DIBH and Patient Selection

As cardiac toxicity due to radiation therapy has a prolonged latency period, no clinical data exists on the effects of DIBH on cardiac morbidity and mortality, and it is currently too soon to tell if it effectively improves these outcomes. Despite this, many dedicated dosimetric comparisons have noted the benefits of DIBH, especially with regards to heart dose parameters. DIBH has been associated with significant improvements in both mean heart doses and mean LAD doses, with respective decreases of 25–67 and 20–73% when comparing the same patients planned with free breathing and DIBH (Table 1). Additionally, real world retrospective data from a large analysis of both community and academic centers also demonstrates that patients treated with DIBH had on average lower heart doses than those treated with free breathing (73).

Studies have noted perfusion defects in those patients who underwent radiation therapy for left-sided breast cancers, with defects correlating to the radiation fields used for treatment (74, 75). Moreover, no perfusion defects were found in patients treated with radiation fields that excluded the heart entirely (76, 77). While a study by Zellars et al. did not find a difference in the incidence of perfusion defects when comparing ABC with free breathing, excluding the heart completely from the treatment field was not required in this study (78). Despite differences in outcomes, the combined data may suggest that DIBH can potentially reduce cardiac toxicity from radiation therapy. It appears that the benefit of DIBH for heart dose spans across 3D and IMRT treatments and those patients undergoing nodal and/or CW irradiation (79). An example of heart movement with respect to chest wall tangent position with free breathing and vDIBH, along with accompanying dose volume histogram (DVH) comparing mean heart and LAD doses for both a standard free breathing and vDIBH plan for a patient receiving CW and regional nodal irradiation is shown in Figures 1G–I.

Although DIBH is well tolerated by most patients, patients should be screened and carefully selected after taking into account factors such as ability to tolerate the technique, cost, patient convenience, and potential benefit based on size, location, and type of tumor. As noted previously, patients with left-sided breast cancers appear to have increased cardiac mortality simply due to the proximity of the target to the heart, therefore, it is likely these patients that would benefit most from the DIBH technique. Based on this, many centers currently only offer DIBH to patients with left-sided disease.

Patients with right-sided breast cancers may also benefit from DIBH, largely due to decreased ipsilateral and total lung doses associated with use of DIBH in patients receiving IMC radiation (60). Additionally, DIBH has been shown to more significantly decrease the heart dose in women receiving nodal irradiation to the IMC (56), largely due to the increased heart dose associated with IMC treatment when compared to treatment of the breast and CW alone (18). While the cardiac toxicity associated with IMC irradiation is worse with left-sided disease, the risk applies to patients with both left- and right-sided disease due to proximity of the target to the heart. DIBH use in right-sided patients can also result in lowered ipsilateral lung and liver doses (80). Hence, these patients with right-sided disease and concern for lung dose, as well as patients undergoing IMC irradiation should also be considered for DIBH. Tolerability of the technique, as noted above, should also be a consideration. While the technique is well tolerated overall, DIBH treatment can take longer when compared to a standard free breathing technique, and patients should be able to lie comfortably flat on their back for the duration of treatment.

One also questions whether patient-specific factors such as anatomical variations should play into patient selection for DIBH. Maximum heart distance, which is defined as the maximum distance within the radiation field between the anterior edge of the heart contour and the posterior border of the tangent field, has been shown to correlate with the mean heart dose (81, 82). A study by Rochet et al. also found that the parasagittal cardiac contact distance, as measured in free breathing, can be used to predict heart dose, with longer distances correlating with higher doses (53). Lee et al. noted similar findings with heart contact both in the axial and parasagittal planes being strongly correlated with mean heart dose (83). These studies suggest that patients with these anatomic factors may benefit most from DIBH. Therefore, consideration should be made to measure these distances on the free-breathing scan and use them as predictive factors for patients who should be selected for DIBH. Within patients who undergo DIBH, patients who have the greatest change in lung volumes between DIBH and free-breathing scans tend to derive the greatest benefit from DIBH in terms of heart dose (54). Despite this, at least 75% of patients who undergo DIBH appear to derive benefit due to decreased heart dose (53). Other anatomic factors can also influence cardiac doses. A more pendulous left breast could lower the inferior border of the tangential fields and, therefore, increase the heart dose. In addition, a tumor bed situated more posterior may cause the dose from the boost fields to impact heart doses. A rapid planning method to generate a free-breathing plan in 9 min was introduced by Wang et al. (41) to help with DIBH patient selection. If the free-breathing plan resulted in a heart V50% greater than 10 cm^3^, the patient was considered to have unfavorable cardiac anatomy and DIBH planning was recommended.

Treatment planning for DIBH plans is not unlike planning for free-breathing breast plans. Much like free breathing, treatment modalities include 3D radiation therapy, IMRT, as well as arc therapy (34, 48, 50). Some studies have reported that wide tangents, when combined with DIBH, may reduce cardiac dose even further (34), and the technique has been favored by many with regard to treatment of the IMCs (84, 85). In addition, the wide tangent technique achieved superior IMC dose coverage compared to the traditional photon–electron technique (86, 87), which makes wide tangents the favorable technique in DIBH planning for IMC irradiation. However, a recent paper from Borm et al. evaluated the dosimetric impact of DIBH on the axillary lymph nodes (88). In this study, the authors demonstrated that the axillary nodes move with DIBH, and the magnitude of that movement is significantly different than the movement of the lumpectomy cavity and the breast. This can result in a reduction of incidental dose to the axilla, with the mean dose to the level 1 axilla reduced by roughly 10% (88). For patients with a positive sentinel node biopsy who forgo axillary dissection, this incidental radiation dose to axillary level 1 may contribute to the low risk of axillary recurrence risk observed in patients treated in this manner (89, 90). Consequently, if therapeutic dose to the level 1 axilla is desired, these authors recommend delineating that region as a target and designing fields appropriately to cover the target.

Another consideration with DIBH is variability in organ and structure position both within and between treatments. The breast itself has been found to have both inter- and intra-fractional reproducibility during DIBH treatments (68, 91), with minimal shifts when aligning to bony anatomy (37). Reassuringly, the interfraction positional variability of the LAD is comparable between DIBH and free breathing (92), while the LAD displacement at DIBH has been noted to be more variable (93). The LAD displacement could result in different cardiac doses than predicted with planning, with significant increases in heart dose when a field sets up too “deep,” but small decreases in heart dose when a field is too “shallow.” The results may be significant due to steep dose gradients at the field edge. As a result, even if the geometric setup errors average out over the course of treatment, the dosimetric changes may not average out. Instead, the LAD dose may be systematically higher than seen at planning. Due to the potential importance of the dose received by the LAD, especially with studies noting cardiac perfusion defects as above, a planning organ at risk volume (PRV) with a margin of 0.5 cm is recommended for the LAD contour (93).

## Additional Heart Sparing Techniques

As noted above, there are now multiple techniques that can be used for cardiac sparing, including prone positioning, IMRT, and proton radiotherapy. Lateral decubitus positioning can also be utilized (94). Each of these techniques can be used in conjunction with, or as an alternative to, DIBH. In order to select the optimal technique, comparisons must be made of each treatment combination. Treatment in the prone position takes advantage of gravity in order to pull the breast away from the CW. This allows the utilization of shallow tangent beams. Dosimetric studies uniformally find that the prone position reduces lung dose from whole breast radiotherapy (22). Most dosimetric series of left breast irradiation find that heart dose is lower in the prone position as compared to supine free breathing, however, because in certain patients the heart can fall forward with gravity (95), the benefit in cardiac dose sparing with prone positioning can vary (22, 96, 97). For this reason, coverage of the IMC can be prohibitive in the prone position due to heart dose. However, nodal radiotherapy in the prone position is feasible if the IMC is to be omitted (98, 99). In dosimetric comparisons of the prone position versus supine DIBH, lung dose was lower in the prone position while heart dose was lower with supine DIBH (27, 100). A group in Belgium has explored by combining these techniques (28, 29). Early data suggest that prone DIBH combines the best of these two techniques yielding very low lung dose (similar to the prone position) and very low heart dose (similar to DIBH). Whether the small incremental improvements in heart and lung dose provided by prone DIBH yield clinical advantages is not yet known.

Proton therapy is another breast cancer radiation modality used to spare heart radiation exposure, taking advantage of the dosimetric properties of protons to reduce cardiac doses. Recent series have shown remarkably low cardiac doses with proton therapy (101). Comparisons of protons at free-breathing versus photons with DIBH have shown that both techniques yield remarkably low heart doses, although proton plans appear to deliver lower mean heart dose and lower dose to the LAD (102). Whether the very small differences in mean heart dose between protons and photons at DIBH will be clinically relevant is in question. Combination series of protons delivered at DIBH have appeared to show no significant improvements over protons at free breathing (103). Notably, the breast contour can evolve significant during radiotherapy due to reabsorption of the seroma or breast edema of shrinkage (104). Proton dosimetry is somewhat less robust to changes in breast size that can occur over the course of treatment as compared to photon plans. As a result, imaging during the RT course and replanning in the event of a contour change, could be considered. Whether the very small differences in mean heart dose between photons using DIBH and protons, and whether the physical and biological uncertainties associated with protons, will be clinically relevant is in question. These questions may be answered by the currently accruing PCORI RADCOMP trial (105). This trial aims to enroll 1,716 patients receiving radiotherapy to the breast or CW in conjunction with the internal mammary nodes and randomize them to radiotherapy with either protons or photons. The primary endpoints of this trial are cardiac events and cancer control events.

There is not yet consensus in the field coalescing around an optimal technique or combination of techniques for cardiac sparing, as evidenced by ongoing investigations and the PICORI RADCOMP trial. We currently favor the use of the prone position or supine DIBH when treating the intact left breast. When treating the left breast or CW and regional nodes, we typically create a plan using 3D conformal radiation therapy with the patient supine at DIBH, and use this approach to treat the majority of patients. IMRT at DIBH is considered in the event that normal tissue constraints are not met with a 3D conformal radiation therapy approach.

## Conclusion

Radiation therapy for breast cancer has been proven to improve both local control and overall survival in patients; however, cardiac toxicity associated with radiation dose to the heart may partially negate these effects. Improvements and advances in radiation technology and delivery over time have led to dramatic decreases in heart doses for those receiving breast cancer radiation. DIBH is an important tool for cardiac sparing and has been reproducibly associated with a reduction of mean heart dose. This benefit is the greatest in those patients with left-sided disease and those receiving IMC irradiation. DIBH has also been shown to decrease dose to the lungs. Further studies aimed to enhance DIBH techniques and to optimize patient selection for DIBH are ongoing.

## Author Contributions

CB, AC, ND, AT, and JS conceived of the manuscript, drafted the manuscript, and approved of the final version to be published.

## Conflict of Interest Statement

The authors declare that the research was conducted in the absence of any commercial or financial relationships that could be construed as a potential conflict of interest.
